# Mirtazapine and Slow-Wave Sleep: A Focused Narrative Review of Human Polysomnographic Evidence

**DOI:** 10.7759/cureus.114784

**Published:** 2026-08-19

**Authors:** Binh Pham

**Affiliations:** 1 Sleep Medicine, Southwest Healthcare Medical Education Consortium, Temecula, USA

**Keywords:** antidepressants, histamine h1 receptor, insomnia, mirtazapine, nassa, polysomnography (psg), rem and nrem sleep, sleep architecture, sleep pharmacology, slow-wave sleep

## Abstract

Sleep disturbance is a common feature of depressive disorders and may be influenced by antidepressant-associated alterations in sleep architecture. Mirtazapine is a noradrenergic and specific serotonergic antidepressant with a distinctive pharmacologic profile involving histamine H1 receptor antagonism, alpha-2 adrenergic antagonism, and serotonergic receptor modulation, which may contribute to its effects on sleep regulation. This focused narrative review examines the neurobiological mechanisms through which mirtazapine may influence sleep architecture and summarizes available human polysomnographic (PSG) evidence, with particular emphasis on N3 sleep and slow-wave sleep-related measures. Current evidence suggests that mirtazapine can produce measurable changes in objective sleep architecture; however, findings specifically regarding N3 sleep and slow-wave sleep remain preliminary and mixed. Available studies are limited by small sample sizes, heterogeneous populations, variable treatment durations, differences in comparator conditions, and methodological variability. Although selected studies have reported increases in slow-wave sleep measures or N3 sleep, available evidence does not establish a consistent dose-response relationship or demonstrate that sedative effects directly translate into enhancement of slow-wave sleep. Mechanistic evidence supports several biologically plausible pathways through which mirtazapine may influence sleep regulation, including histaminergic, adrenergic, and serotonergic effects; however, human PSG studies evaluate the integrated effects of the complete medication and cannot establish receptor-specific mechanisms responsible for observed sleep changes. Future studies using standardized PSG protocols, quantitative electroencephalography (EEG) analysis, larger samples, longer treatment durations, and direct dose comparisons are needed to clarify whether mirtazapine-associated alterations in sleep architecture contribute meaningfully to clinical outcomes.

## Introduction and background

Sleep is a highly regulated neurobiological process essential for normal physiologic function and overall health [1,2]. Mirtazapine is commonly used in clinical practice for depressive disorders accompanied by insomnia symptoms because of its sedative properties; however, sedation, reduced sleep latency, and improved sleep continuity should not be considered equivalent to enhancement of slow-wave sleep or normalization of sleep architecture. Normal sleep consists of recurring cycles of non-rapid eye movement (NREM) and rapid eye movement (REM) sleep, with NREM sleep progressing through stages N1, N2, and N3 [1,2]. Among these stages, N3 sleep represents the deepest stage of physiologic sleep and is defined by standardized polysomnographic scoring criteria. N3 sleep is characterized by synchronized high-amplitude, low-frequency delta activity, which reflects increased slow-wave activity on electroencephalographic measures [1,3]. Slow-wave sleep is associated with restorative physiologic processes and has been proposed to contribute to the homeostatic regulation of synaptic strength [2,4]. The classification, principal neurobiology, and potential effects of mirtazapine on normal sleep stages are summarized in Table 1.

**Table 1:** Normal sleep architecture: classification, neurobiology, and potential effects of mirtazapine. The potential effects of mirtazapine summarized in this table are qualitative and represent concepts evaluated in the broader pharmacologic and human polysomnographic literature discussed throughout the manuscript. Reported effects may vary according to dosage, treatment duration, study population, baseline sleep characteristics, concomitant medications, and polysomnographic methodology. N3 sleep and slow-wave activity represent related but distinct outcomes: N3 sleep refers to a visually scored sleep stage based on electroencephalographic criteria, whereas slow-wave activity reflects quantitative EEG spectral measures of delta-frequency activity. Sedation, reduced sleep latency, or improved sleep continuity should not be considered equivalent to enhancement of slow-wave sleep or normalization of sleep architecture. EEG: electroencephalography; NREM: non-rapid eye movement; REM: rapid eye movement; PSG: polysomnography; N3: stage N3 sleep.

Sleep stage	Principal characteristics	Principal neurobiology	Potential effects of mirtazapine
N1 sleep	Transitional stage between wakefulness and sleep characterized by reduced alpha activity, increased theta activity, and decreased responsiveness to external stimuli [1,2]	Represents the transition from wakefulness to sleep through progressive reduction of arousal-promoting activity and engagement of sleep-promoting networks [1,2]	Sedative effects of mirtazapine may influence sleep initiation and sleep propensity; however, improvements in sleep onset or sedation should not be interpreted as evidence of enhanced slow-wave sleep
N2 sleep	Intermediate stage of NREM sleep characterized by sleep spindles and K-complexes, reflecting stable sleep and reduced sensory processing [1,2]	Generated through coordinated thalamocortical interactions that stabilize sleep and regulate sensory gating [1,3]	Potential effects of mirtazapine on N2 sleep architecture are discussed within the broader context of antidepressant pharmacology and human polysomnographic studies
N3 sleep (slow-wave sleep)	Deepest stage of NREM sleep characterized by high-amplitude, low-frequency delta oscillations and increased arousal threshold [1-3]	Regulated by synchronized cortical and thalamic oscillations, homeostatic sleep pressure, and coordinated activity of sleep-regulatory networks [3-5]	Potential effects of mirtazapine on N3 sleep and slow-wave sleep measures remain under investigation. Whether modulation of these parameters translates into clinical benefit remains uncertain
REM sleep	Characterized by cortical activation, rapid eye movements, muscle atonia, and wake-like electroencephalographic activity [1,2]	Controlled by interactions among REM-promoting brainstem circuits, cholinergic activity, and monoaminergic regulation of REM suppression [1,2]	Potential effects of mirtazapine on REM sleep are considered within the broader context of antidepressant-related modulation of sleep architecture

Slow-wave sleep represents an important component of normal sleep architecture and has been associated with processes including memory consolidation, synaptic regulation, and optimization of neural function. However, these associations do not establish that pharmacologically increasing slow-wave sleep necessarily produces corresponding improvements in clinical outcomes. The clinical relevance of slow-wave sleep has gained increasing attention because this sleep stage declines with aging and may be disrupted across neuropsychiatric disorders. Therefore, potential effects of mirtazapine on slow-wave sleep should be considered within the broader framework of antidepressant efficacy, sleep quality, daytime functioning, and medication tolerability rather than as an isolated therapeutic endpoint.

Slow-wave sleep is generated through coordinated interactions among cortical and thalamic networks, sleep-promoting hypothalamic pathways, and ascending arousal systems that regulate transitions between wakefulness and sleep [1,6]. Homeostatic sleep pressure accumulated during wakefulness contributes to the expression of slow-wave activity during subsequent sleep, while reduced activity of wake-promoting systems and increased activity of sleep-promoting pathways facilitate stable NREM sleep [4,5,7]. Because many psychotropic medications target neurotransmitter systems involved in sleep-wake regulation, they may modify objective sleep architecture in addition to producing subjective effects on sleep quality and sedation [8].

The effects of antidepressants on sleep architecture have become increasingly relevant because sleep disturbances are common among patients with depressive disorders and may persist despite improvement in mood symptoms [8]. Although many antidepressants improve subjective sleep quality, their effects on objectively measured sleep architecture differ substantially across pharmacologic classes [8,9]. Suppression of REM sleep is a relatively consistent finding with numerous antidepressants, whereas effects on slow-wave sleep appear more variable and may depend on medication-specific receptor-binding characteristics rather than representing a class-wide phenomenon [8,9]. These differences highlight the importance of human polysomnography (PSG) for distinguishing improvements in sleep continuity from genuine alterations in physiologic sleep architecture [8].

Among currently available antidepressants, mirtazapine is classified as a noradrenergic and specific serotonergic antidepressant (NaSSA) and possesses a pharmacologic profile distinct from monoamine transporter inhibitors [10,11]. Rather than inhibiting serotonin or norepinephrine reuptake, mirtazapine enhances monoaminergic neurotransmission primarily through antagonism of presynaptic alpha-2 adrenergic autoreceptors and heteroreceptors while simultaneously antagonizing histamine H1 receptors and several serotonin (5-hydroxytryptamine (5-HT)) receptor subtypes, including 5-HT2A, 5-HT2C, and 5-HT3 [10-12]. Histamine H1 receptor antagonism is generally considered a major contributor to the sedative properties of mirtazapine, whereas downstream effects on sleep architecture may reflect the combined influence of multiple receptor systems, including serotonergic modulation. Although serotonergic receptor modulation, including 5-HT2A antagonism, has been proposed as a potential contributor to sleep architecture changes, human polysomnographic studies cannot determine whether specific receptor mechanisms are responsible for observed changes in slow-wave sleep because mirtazapine simultaneously acts at multiple pharmacologic targets [10,12]. Therefore, mirtazapine provides a unique pharmacologic model for examining whether receptor-specific mechanisms may influence objectively measured sleep architecture beyond subjective sedative effects.

Although several human polysomnographic studies have evaluated the effects of mirtazapine on sleep architecture, including studies by Ruigt et al., Winokur et al., Aslan et al., Schittecatte et al., Shen et al., and Mi et al., the available literature remains limited and heterogeneous [13-18]. Differences in study populations, treatment duration, dosing strategies, and reported outcomes have complicated direct comparisons across studies [13-18]. Consequently, whether mirtazapine consistently enhances slow-wave sleep remains uncertain. This focused narrative review summarizes the neurobiology of slow-wave sleep, examines pharmacologic mechanisms through which mirtazapine may influence sleep architecture, critically evaluates available human PSG evidence, and discusses potential clinical implications and future directions for research.

## Review

Literature search strategy

A focused narrative literature review was conducted to identify relevant evidence regarding the effects of mirtazapine on sleep architecture, with particular emphasis on human polysomnographic (PSG) findings related to slow-wave sleep and N3 sleep. Electronic searches were performed using PubMed and Google Scholar in August 2026 to identify relevant English-language publications. No date restrictions were applied. The PubMed search strategy included combinations of the following terms: ("mirtazapine" OR "Org 3770") AND ("sleep architecture" OR "polysomnography" OR "polysomnographic" OR "sleep stages" OR "slow-wave sleep" OR "slow wave sleep" OR "N3 sleep" OR "non-rapid eye movement sleep"). Additional related keyword combinations were used in Google Scholar to identify potentially relevant publications not captured by the primary database search. Reference lists of relevant articles and review papers were manually screened for additional eligible studies. Retrieved records were reviewed for duplicate publications prior to final study selection.

Studies were considered eligible for inclusion if they met the following criteria: (1) human participants; (2) evaluation of mirtazapine exposure; (3) objective assessment of sleep using polysomnography or laboratory-based sleep measurements; and (4) reporting of sleep architecture outcomes, including NREM sleep stages, N3 sleep, slow-wave sleep, slow-wave activity, or related PSG parameters. Studies were excluded if they involved animal models, in vitro investigations, lacked objective sleep measurements, did not evaluate mirtazapine, or did not report clinically relevant sleep architecture outcomes.

Relevance and methodological quality were assessed qualitatively by the authors according to study design, sample characteristics, presence of appropriate controls or comparators when applicable, objective assessment methods, relevance of measured outcomes, and limitations that could influence interpretation. Because this manuscript represents a focused narrative review rather than a systematic review, no formal risk-of-bias assessment tool was applied. Potential limitations related to study heterogeneity and selection bias were considered during interpretation of the available evidence.

Evidence was weighted according to its direct relevance to the central question of whether mirtazapine alters human slow-wave sleep. Human PSG studies were considered the primary evidence base because they directly evaluate objective sleep architecture and slow-wave sleep-related parameters. Non-PSG human studies were included when they provided relevant clinical context but were not considered direct evidence of changes in sleep architecture. Animal studies and mechanistic/receptor studies were included to provide biological plausibility and contextual understanding of potential pathways through which mirtazapine may influence sleep regulation; however, these studies were not interpreted as evidence of demonstrated effects on human slow-wave sleep.

Relevant publications were evaluated based on title and abstract relevance, followed by full-text review when appropriate. Studies were excluded if they did not include human PSG evaluation, did not evaluate mirtazapine, or did not report relevant objective sleep architecture outcomes. Characteristics and principal findings of included human PSG studies were extracted, including study population, sample size, mirtazapine dose, treatment duration, comparator conditions, PSG endpoints, and major findings.

Because this manuscript represents a focused narrative review rather than a systematic review, formal meta-analysis, quantitative synthesis, and PRISMA-based flow analysis were not performed. Quantitative pooling was not considered appropriate because the available human PSG studies are limited in number and demonstrate substantial heterogeneity in study populations, psychiatric diagnoses, mirtazapine dosing strategies, treatment durations, comparator conditions, PSG methodology, and reported sleep architecture outcomes. The available literature was critically evaluated with consideration of study design characteristics, methodological limitations, and consistency of reported findings. Limitations related to study heterogeneity, selection bias, and narrative review methodology are acknowledged.

Neurobiological regulation of slow-wave sleep

Slow-wave sleep represents the deepest stage of non-rapid eye movement (NREM) sleep and depends on coordinated interactions among cortical and thalamic networks that generate synchronized oscillatory activity, together with hypothalamic and brainstem systems that regulate transitions between wakefulness and sleep [1,3,6]. Unlike rapid eye movement (REM) sleep, which is characterized by cortical activation and desynchronized electroencephalographic activity, N3 sleep is a polysomnographically defined sleep stage characterized by increased electroencephalographic slow-wave activity, including synchronized high-amplitude, low-frequency delta oscillations arising from coordinated cortical and thalamocortical network interactions [1,3]. Although N3 sleep and slow-wave activity are closely related, they represent distinct measures, with N3 referring to a scored sleep stage and slow-wave activity reflecting an electrophysiologic measure of delta-range EEG activity. The expression of slow-wave sleep is strongly influenced by homeostatic sleep pressure, which accumulates during wakefulness and contributes to increased slow-wave activity during subsequent sleep [4,5]. Together, these neurophysiologic processes provide the framework through which endogenous regulatory systems influence sleep depth and stability.

The electrophysiologic characteristics of N3 sleep arise from reciprocal communication between cortical pyramidal neurons and thalamic relay neurons, generating synchronized oscillatory activity that contributes to the large-amplitude delta waves observed during deep NREM sleep [1,3]. Increased neuronal synchronization is associated with reduced responsiveness to external sensory input and higher arousal thresholds, reflecting the reduced environmental responsiveness characteristic of deep NREM sleep [1]. At the systems level, transitions between wakefulness and sleep are stabilized through reciprocal interactions between sleep-promoting hypothalamic pathways and ascending arousal systems, creating relatively stable behavioral states rather than continuous intermediate levels of arousal [6]. This coordinated network organization supports the physiologic processes associated with slow-wave sleep.

The transition from wakefulness to N3 sleep involves coordinated changes across multiple neurotransmitter systems that regulate arousal and sleep promotion [1,6]. During wakefulness, histaminergic, noradrenergic, serotonergic, and cholinergic systems contribute to cortical activation and behavioral arousal, whereas activity within these wake-promoting systems declines during NREM sleep [6]. In contrast, gamma-aminobutyric acid (GABA)-ergic neurons within the ventrolateral preoptic nucleus promote sleep initiation and maintenance through inhibition of major components of the ascending arousal system [6]. In parallel, adenosine accumulation during prolonged wakefulness has been implicated in homeostatic sleep pressure and is associated with subsequent increases in slow-wave activity during sleep [7]. Collectively, these interacting neurotransmitters and homeostatic signaling systems contribute to the timing, depth, and expression of slow-wave sleep.

The neurobiological regulation of slow-wave sleep depends on the dynamic interplay between distributed neural circuits, homeostatic processes, and multiple neurotransmitter systems, making sleep architecture responsive to pharmacologic modulation [1,8]. Medications that modify neurotransmitter systems involved in sleep-wake regulation may have the potential to influence objective sleep architecture; however, the specific effects of individual medications depend on their receptor-binding profiles, pharmacodynamic properties, and direct evaluation through objective sleep assessments [8]. Consequently, medication-associated changes in slow-wave sleep should not be inferred solely from receptor pharmacology but require confirmation through PSG-based outcomes. Among currently available antidepressants, mirtazapine possesses a distinctive receptor-binding profile that provides a biologically plausible framework for examining potential effects on slow-wave sleep, forming the mechanistic foundation for the pharmacologic discussion that follows.

Pharmacology of mirtazapine relevant to slow-wave sleep

Mirtazapine is classified as a noradrenergic and specific serotonergic antidepressant (NaSSA) and possesses a pharmacologic profile that differs substantially from many conventional antidepressants [10-12]. Unlike selective serotonin reuptake inhibitors (SSRIs) and serotonin-norepinephrine reuptake inhibitors (SNRIs), mirtazapine does not inhibit monoamine transporters but instead enhances monoaminergic neurotransmission through receptor-mediated mechanisms [10,11]. This distinct pharmacologic profile provides a biologically plausible framework for examining potential effects on sleep architecture; however, differences in antidepressant mechanism alone do not establish differential effects on N3 sleep or slow-wave activity. Mirtazapine's pharmacologic actions include antagonism of presynaptic alpha-2 adrenergic autoreceptors and heteroreceptors together with potent antagonism of histamine H1 receptors and several serotonin (5-hydroxytryptamine (5-HT)) receptor subtypes, including 5-HT2A, 5-HT2C, and 5-HT3 [10,12]. Because these receptor systems participate in the regulation of arousal, cortical excitability, and sleep-wake regulation, mirtazapine has attracted considerable interest as a potential modulator of sleep architecture.

Among the pharmacologic properties of mirtazapine, antagonism of histamine H1 receptors is considered a major contributor to its sedative effects [10,12,19]. Histaminergic neurons arising from the tuberomammillary nucleus constitute a major component of the ascending arousal system and promote cortical activation during wakefulness [1,6]. Blockade of H1 receptors reduces wake-promoting histaminergic signaling and may facilitate sleep initiation and improvements in sleep continuity [1,10]. Clinically, lower doses of mirtazapine are sometimes used off-label to address insomnia symptoms because of its prominent histamine H1 receptor antagonism and associated sedative effects. A commonly proposed explanation for greater sedation observed by some patients at lower doses is that H1 receptor-mediated effects may be relatively more prominent at lower doses, whereas increased noradrenergic activity with alpha-2 adrenergic antagonism at higher doses has been hypothesized to partially counterbalance sedation. However, this dose-dependent receptor-occupancy model remains incompletely established, and clinical studies do not consistently demonstrate that higher doses of mirtazapine are less sedating than lower doses [10,12,19]. Importantly, subjective sedation, reduced sleep latency, and improved sleep continuity should not be interpreted as evidence of enhanced slow-wave sleep without objective PSG confirmation.

Mirtazapine exerts its antidepressant effects through antagonism of presynaptic alpha-2 adrenergic autoreceptors and heteroreceptors, thereby enhancing both noradrenergic and serotonergic neurotransmission [10-12]. Blockade of alpha-2 autoreceptors increases norepinephrine release, whereas antagonism of alpha-2 heteroreceptors facilitates serotonin release through disinhibition of serotonergic neurons [10,11]. Although these mechanisms primarily contribute to antidepressant efficacy, they also modify neurotransmitter systems involved in sleep-wake regulation [1,8]. Alterations in monoaminergic signaling may indirectly influence slow-wave sleep through downstream effects on hypothalamic and thalamocortical circuits involved in transitions between wakefulness and stable NREM sleep. However, evidence supporting a direct role of alpha-2 adrenergic antagonism in promoting N3 sleep remains limited, and any contribution is likely secondary to broader modulation of sleep-regulatory networks [8,10-12].

Mirtazapine also exhibits potent antagonism of 5-HT2A, 5-HT2C, and 5-HT3 receptors, although the relevance of these receptor subtypes to slow-wave sleep differs substantially [10-12]. Among these, 5-HT2A receptor antagonism has attracted particular interest because 5-HT modulation of cortical excitability may influence sleep architecture [8,10]. By altering 5-HT signaling within sleep-regulatory networks, 5-HT2A receptor antagonism represents one biologically plausible hypothesis through which mirtazapine may influence slow-wave sleep-related measures. However, human PSG studies cannot determine whether 5-HT2A antagonism specifically mediates observed changes in N3 sleep or slow-wave activity because mirtazapine simultaneously acts at multiple receptor systems. The potential contributions of 5-HT2C and 5-HT3 receptor antagonism to slow-wave sleep remain less clearly defined and are supported primarily by indirect mechanistic evidence rather than receptor-specific polysomnographic findings [11,12].

Collectively, the pharmacologic profile of mirtazapine provides several biologically plausible pathways through which the medication may influence sleep regulation and sleep architecture [10-12]. Histamine H1 receptor antagonism provides a plausible explanation for sedative effects and sleep initiation, whereas serotonergic and adrenergic modulation may contribute to observed changes in objective sleep architecture [10-12]. However, these proposed mechanisms should be interpreted with caution because whole-drug PSG studies assess the integrated effects of multiple simultaneous receptor actions and cannot establish receptor-specific causal relationships. In particular, observed changes in N3 sleep or slow-wave sleep measures cannot be definitively attributed to modulation of histamine H1, alpha-2 adrenergic, or serotonergic receptors based solely on PSG findings [10-18]. These mechanisms likely interact rather than function independently, and available human studies are insufficient to determine whether any individual receptor pathway is primarily responsible for observed changes in sleep architecture [13-18]. The established pharmacologic actions of mirtazapine and their proposed relationships to sleep regulation and sleep architecture, including potential but unconfirmed effects on slow-wave sleep measures, are summarized in Table 2.

**Table 2:** Pharmacologic targets of mirtazapine and proposed relationships with sleep regulation. Mirtazapine’s effects on sleep architecture likely reflect the combined influence of multiple receptor actions rather than a single pharmacologic pathway. Individual receptor mechanisms represent biologically plausible hypotheses based on known pharmacology and sleep-regulatory pathways; however, available human polysomnographic studies evaluate the effects of the complete drug molecule and cannot establish receptor-specific causal relationships. Therefore, proposed associations between individual receptor targets and slow-wave sleep should be interpreted as mechanistic hypotheses rather than demonstrated effects. 5-HT: 5-hydroxytryptamine (serotonin); H1: histamine H1 receptor; NaSSA: noradrenergic and specific serotonergic antidepressant; N3: stage N3 sleep; PSG: polysomnography.

Pharmacologic target	Established pharmacologic effects of mirtazapine	Potential relationship to sleep regulation	Evidence regarding slow-wave sleep
Histamine H1 receptor antagonism	Mirtazapine exhibits potent H1 receptor antagonism, contributing substantially to its sedative properties and effects on arousal regulation [10,12]	Histaminergic neurons arising from the tuberomammillary nucleus contribute to ascending arousal signaling; H1 receptor blockade may reduce wake-promoting activity and facilitate sleep initiation [1,6]	Sedative effects and improvements in sleep continuity should not be interpreted as equivalent to enhancement of N3 sleep or slow-wave activity. Although H1 receptor antagonism provides a plausible explanation for sedation, its direct contribution to slow-wave sleep remains incompletely characterized [8,10]
Alpha-2 adrenergic autoreceptor and heteroreceptor antagonism	Mirtazapine antagonizes presynaptic alpha-2 adrenergic autoreceptors and heteroreceptors, increasing noradrenergic and serotonergic neurotransmission [10-12]	Increased monoaminergic signaling may influence sleep-wake regulation through downstream effects on arousal systems and sleep-promoting networks [1,6,8]	A direct role of alpha-2 adrenergic antagonism in promoting N3 sleep has not been established. Any contribution is likely related to broader network-level modulation rather than isolated receptor activity [8,10-12]
5-HT2A receptor antagonism	Mirtazapine antagonizes 5-HT2A receptors as part of its broader serotonin (5-hydroxytryptamine (5-HT)) receptor profile [10-12]	5-HT2A-mediated regulation of cortical excitability and sleep architecture provides a biologically plausible mechanism through which serotonergic modulation may influence NREM sleep [8,10]	5-HT2A antagonism represents a biologically plausible hypothesis for influencing sleep architecture; however, human PSG studies cannot determine whether 5-HT2A antagonism specifically mediates observed changes in N3 sleep or slow-wave activity because mirtazapine acts across multiple receptor systems [10-12]
5-HT2C receptor antagonism	Mirtazapine antagonizes 5-HT2C receptors as part of its overall receptor-mediated pharmacologic profile [10-12]	5-HT2C signaling may influence monoaminergic regulation and sleep-wake pathways [8,10]	The contribution of 5-HT2C antagonism to slow-wave sleep remains uncertain and is supported primarily by indirect mechanistic evidence rather than receptor-specific PSG findings [11,12]
5-HT3 receptor antagonism	Mirtazapine antagonizes 5-HT3 receptors, reducing signaling through this serotonergic receptor subtype [10-12]	5-HT3 receptor modulation may influence interactions among neurotransmitter systems involved in sleep regulation [11,12]	A direct relationship between 5-HT3 antagonism and slow-wave sleep enhancement has not been demonstrated in human PSG studies. Available evidence remains limited to indirect pharmacologic considerations [11,12]
Integrated receptor effects (NaSSA profile)	Mirtazapine produces combined effects through histaminergic, adrenergic, and serotonin receptor modulation rather than through a single pharmacologic pathway [10-12]	Sleep architecture effects likely reflect interactions among multiple sleep-regulatory systems rather than isolated receptor actions [1,6,8]	Available human PSG evidence supports possible effects on selected sleep architecture measures; however, whole-drug PSG observations cannot establish receptor-specific causal mechanisms underlying these changes [13-18]

Human polysomnographic evidence

Although mirtazapine possesses several pharmacologic properties that may influence sleep regulation, interpretation of its effects on sleep architecture requires consideration of both medication-related effects and baseline abnormalities associated with psychiatric illness. Polysomnography (PSG) provides a standardized assessment of sleep architecture, including visually scored sleep stages such as N1, N2, and N3 sleep, as well as other objective sleep parameters, including sleep latency, total sleep time, sleep efficiency, and rapid eye movement (REM)-related measures according to established scoring criteria [20]. Major depressive disorder is independently associated with disrupted sleep architecture, including impaired sleep continuity, alterations in NREM sleep organization, abnormalities in REM sleep regulation, and broader disturbances in sleep regulation [21]. Importantly, N3 sleep, slow-wave sleep, and slow-wave activity represent related but distinct concepts: N3 is a visually scored sleep stage defined by EEG criteria; slow-wave sleep generally refers to conventional PSG-derived measures of deep NREM sleep corresponding to N3; whereas slow-wave activity reflects quantitative EEG spectral measures of delta-frequency activity. The available human PSG studies evaluating mirtazapine primarily assessed conventional sleep architecture variables, including N3 or slow-wave sleep duration/percentage, sleep continuity parameters, and REM-related outcomes, rather than formal EEG spectral measures of slow-wave activity.

Interpretation of these findings also requires consideration of study population and comparator design. Studies conducted in healthy volunteers provide a more direct assessment of medication-related effects on sleep physiology because they are less influenced by illness-associated abnormalities; however, their findings may have limited generalizability to patients with depressive disorders. Conversely, studies conducted in patients with major depressive disorder provide greater clinical relevance but require cautious interpretation because depression itself may contribute to baseline abnormalities in sleep architecture [21]. Furthermore, available studies used different comparator approaches, including placebo-controlled investigations in healthy volunteers and treatment-based evaluations in depressed populations without healthy control groups or placebo comparators. Therefore, PSG changes observed during mirtazapine treatment may reflect direct pharmacologic effects on sleep-regulatory systems; however, in studies involving patients with depression, improvement of underlying depression-associated sleep disruption represents a possible contributing factor that cannot be established as the mechanism responsible for observed PSG changes.

Ruigt et al. evaluated the effects of Org 3770, the investigational compound later developed as mirtazapine, on human sleep in a double-blind, placebo-controlled crossover polysomnographic study of six young healthy male volunteers [13]. Participants received a single 30 mg oral dose of Org 3770 two hours before bedtime, allowing assessment of acute pharmacologic effects on sleep physiology without the potential confounding influence of depressive illness. Compared with placebo, Org 3770 shortened sleep onset latency, reduced bedtime waking and stage 1 sleep, and increased deep slow-wave sleep (stages 3 and 4). The study also demonstrated increased REM sleep latency and modest reductions in awakenings during REM sleep. These findings provide early evidence that the compound later developed as mirtazapine can directly influence objective sleep architecture; however, interpretation is limited by the small sample size, acute single-dose design, and healthy volunteer population. Additionally, because mirtazapine acts across multiple receptor systems, these findings cannot establish a specific receptor mechanism responsible for observed changes in sleep architecture.

Winokur et al. examined the acute effects of mirtazapine on sleep continuity and sleep architecture in six patients with major depressive disorder using PSG recordings obtained at baseline and after one and two weeks of open-label treatment with mirtazapine (15 mg nightly during week one and 30 mg nightly during week two) [14]. Mirtazapine treatment was associated with reduced sleep latency and increased total sleep time and sleep efficiency, while REM sleep parameters were not significantly altered. The study primarily demonstrated improvements in sleep continuity measures rather than definitive changes in N3 sleep or slow-wave sleep parameters. Although the authors concluded that mirtazapine improved sleep continuity without major disruption of sleep architecture, the small sample size and open-label design limited conclusions regarding specific effects on slow-wave sleep.

Aslan et al. evaluated the acute effects of mirtazapine on sleep architecture in 20 young healthy volunteers using a randomized, double-blind, placebo-controlled polysomnographic design [15]. Participants received a single 30 mg dose of mirtazapine, allowing evaluation of acute pharmacologic effects on sleep physiology independent of antidepressant response. Compared with placebo, mirtazapine increased slow-wave sleep duration, reduced stage 1 sleep, and improved measures of sleep continuity, while REM sleep parameters were not significantly altered. Because the study was conducted in healthy volunteers without depressive illness, these findings support a direct pharmacologic influence of mirtazapine on sleep architecture without the potential confounding influence of antidepressant response. However, the acute single-dose design and healthy volunteer population limit conclusions regarding longer-term treatment effects and generalizability to patients with depressive disorders.

Schittecatte et al. evaluated the effects of mirtazapine on sleep polygraphic variables in patients with major depression and reported changes in PSG-derived sleep parameters following treatment [16]. These findings contributed to evidence that mirtazapine may influence NREM sleep organization and other measures of sleep architecture in depressed populations. However, interpretation of these findings requires consideration of depression-associated sleep abnormalities and limitations related to study population and treatment methodology.

Shen et al. examined PSG changes in patients with major depressive disorder treated with mirtazapine and reported alterations in objective sleep architecture measures during treatment [17]. These findings provided additional evidence that mirtazapine may modify sleep architecture in clinical populations. However, because the study involved patients with depression and mirtazapine simultaneously affects multiple receptor systems, observed PSG changes cannot be attributed to a specific pharmacologic mechanism.

Mi et al. compared the effects of mirtazapine and agomelatine on sleep disturbances in patients with major depressive disorder and reported changes in PSG-derived sleep parameters during treatment [18]. Notably, increases in N3 sleep were observed early during treatment but were not consistently maintained at later assessment points, suggesting that mirtazapine-associated changes in deep NREM sleep may vary over time. These findings highlight the importance of treatment duration and assessment timing when interpreting effects on sleep architecture.

Although lower-dose mirtazapine is clinically associated with greater sedative effects, often attributed to the prominent contribution of histamine H1 receptor antagonism [10], available human PSG studies have not directly established whether dose influences mirtazapine-associated changes in N3 sleep. Differences in study dose, treatment duration, and methodology limit the ability to determine whether lower doses preferentially enhance slow-wave sleep or whether a consistent dose-response relationship exists [15,19].

Collectively, available human PSG studies suggest that mirtazapine can produce measurable alterations in sleep architecture, including increases in slow-wave sleep measures or N3 sleep reported in selected studies [13,15-18]. However, current evidence remains insufficient to establish a consistent, dose-dependent enhancement of slow-wave sleep because available PSG investigations were not specifically designed to directly compare mirtazapine doses or to evaluate formal dose-response relationships [15-18]. Findings across studies are heterogeneous because of differences in clinical populations, dosing strategies, treatment duration, comparator conditions, and methodology [13-18]. Healthy volunteer studies provide stronger evidence of the direct pharmacologic effects of mirtazapine on sleep physiology, whereas studies in depressive populations offer greater clinical relevance but require cautious interpretation, as observed changes may reflect both medication effects and possible improvement in depression-associated sleep abnormalities [21]. Current evidence does not establish that clinically observed sedative effects translate into sustained enhancement of slow-wave sleep [13-18]. Further controlled PSG investigations using larger samples, standardized methodology, longer treatment durations, and systematic dose-response evaluation are needed.

The characteristics, methodologies, and principal PSG findings of the available human studies evaluating mirtazapine are summarized in Table 3. This summary highlights differences in study populations, treatment approaches, comparator designs, and PSG outcomes that contribute to the heterogeneity of the current evidence base.

**Table 3:** Human polysomnographic studies evaluating the effects of mirtazapine on sleep architecture. Available human PSG studies demonstrate that mirtazapine can influence objective sleep architecture; however, findings remain heterogeneous because of differences in study populations, comparator conditions, dosing strategies, treatment duration, and methodology. Studies in healthy volunteers provide greater evidence regarding direct pharmacologic effects on sleep physiology, whereas studies in patients with depressive disorders provide greater clinical relevance but require cautious interpretation because observed changes may reflect both medication effects and improvement of depression-associated sleep abnormalities. Importantly, available PSG studies primarily evaluated conventional sleep architecture measures, including sleep stages, sleep continuity parameters, and REM-related outcomes, rather than formal quantitative EEG measures of slow-wave activity. Therefore, evidence supporting mirtazapine-associated enhancement of slow-wave sleep remains limited, and receptor-specific mechanisms underlying observed PSG changes cannot be established. EEG: electroencephalography; NREM: non-rapid eye movement; PSG: polysomnography; REM: rapid eye movement; N3: stage N3 sleep.

Study	Population	Design and mirtazapine exposure	Principal PSG findings	Interpretation and limitations
Ruigt et al. (1990) [13]	Six healthy male volunteers	Double-blind, placebo-controlled crossover PSG study evaluating acute administration of Org 3770, the investigational compound later developed as mirtazapine. Participants received a single 30 mg dose before bedtime.	Reduced sleep onset latency, reduced stage 1 sleep and bedtime waking, increased deep slow-wave sleep (stages 3 and 4), increased REM sleep latency, and modest reduction in REM awakenings.	Provides early evidence of direct pharmacologic effects on sleep architecture independent of depressive illness. Limitations include small sample size, acute single-dose exposure, and healthy volunteer population.
Winokur et al. (2000) [14]	Six patients with major depressive disorder and clinically significant sleep disturbance	Open-label PSG study evaluating mirtazapine treatment over two weeks (15 mg nightly during week one and 30 mg nightly during week two).	Reduced sleep latency and increased total sleep time and sleep efficiency. REM sleep parameters were not significantly altered.	Demonstrated improvements primarily in sleep continuity rather than definitive enhancement of N3 sleep or slow-wave sleep. Interpretation limited by small sample size and open-label design.
Aslan et al. (2002) [15]	Twenty young healthy volunteers (mean age 24 years); randomized to placebo (n=10) or mirtazapine (n=10) groups	Randomized, double-blind, placebo-controlled PSG study evaluating acute administration of a single 30 mg dose of mirtazapine. Participants underwent three consecutive laboratory nights: adaptation night, baseline PSG night, and treatment night.	Mirtazapine improved sleep continuity measures compared with placebo, including increased sleep efficiency and reductions in number and duration of awakenings. Slow-wave sleep time increased, stage 1 sleep decreased significantly, and REM sleep variables were not significantly altered.	Provides strong evidence for direct acute pharmacologic effects of mirtazapine on sleep physiology independent of antidepressant response. Limitations include acute single-dose design, small sample size, and limited generalizability to clinical populations.
Schittecatte et al. (2002) [16]	Patients with major depressive disorder	PSG evaluation of sleep polygraphic variables following mirtazapine treatment.	Reported changes in PSG-derived sleep parameters, including alterations in NREM sleep organization and sleep architecture.	Supports potential modulation of sleep architecture in depressed populations; however, findings require cautious interpretation because depression itself may alter baseline sleep architecture.
Shen et al. (2006) [17]	Patients with major depressive disorder	PSG and symptom analysis following mirtazapine treatment.	Demonstrated changes in objective sleep architecture measures during treatment.	Provides evidence of sleep architecture changes in clinical populations; however, PSG findings cannot determine specific receptor mechanisms responsible for observed effects.
Mi et al. (2020) [18]	Patients with major depressive disorder	Comparative PSG study evaluating mirtazapine and agomelatine treatment effects on sleep disturbances.	Mirtazapine was associated with changes in PSG-derived sleep parameters, including increases in N3 sleep early during treatment that were not consistently maintained at later assessment points.	Highlights the importance of treatment duration and assessment timing when interpreting mirtazapine-associated changes in sleep architecture.

Clinical implications

Slow-wave sleep represents a physiologically important component of normal sleep architecture and has been associated with multiple restorative processes, including memory consolidation, synaptic regulation, and broader aspects of neural function [22]. However, these associations do not establish that pharmacologically increasing slow-wave sleep necessarily produces corresponding improvements in clinical outcomes, as alterations in individual sleep parameters may not directly translate into measurable benefits in symptoms, functioning, or long-term health outcomes. The clinical relevance of slow-wave sleep has gained increasing attention because this sleep stage declines with aging and may be disrupted across neuropsychiatric disorders [23,24]. Therefore, potential effects of mirtazapine on slow-wave sleep should be considered within the broader framework of antidepressant efficacy, sleep quality, daytime functioning, and medication tolerability rather than as an isolated therapeutic endpoint.

Mirtazapine occupies a unique clinical position among antidepressants because it is frequently selected for patients with depressive disorders accompanied by insomnia symptoms, reduced appetite, or prominent sleep disturbance due to its combination of antidepressant and sedative properties [8,12,19]. In clinical practice, lower-dose mirtazapine is sometimes used off-label for insomnia symptoms, although its evidence base and clinical role differ from its established indication for the treatment of major depressive disorder. Histamine H1 receptor antagonism is considered an important contributor to its sedative properties, including reduced arousal, increased sleep propensity, and facilitation of sleep initiation [10,12,19]. However, the clinical observation that lower doses may be more sedating should be distinguished from dose-dependent effects on objective sleep architecture.

Although lower-dose mirtazapine is commonly perceived as producing greater sedation, the relationship between dose and sedation is not fully established and is unlikely to represent a simple linear relationship. The proposed contribution of histamine H1 receptor antagonism to mirtazapine-associated sedation provides a biologically plausible explanation for variability in sedative effects across individuals and dosing conditions [10,12]. However, available clinical evidence is insufficient to establish that higher doses are uniformly less sedating or that lower doses consistently produce greater sedative effects across individuals [19]. Therefore, dose-related sedation should be interpreted cautiously and should not be assumed to predict specific effects on sleep physiology.

Importantly, sedation, improvement in sleep continuity, and enhancement of slow-wave sleep represent distinct clinical and physiologic outcomes. Sedative effects may contribute to reduced sleep latency, decreased subjective arousal, and improved perceived sleep quality. Objective PSG measures of sleep continuity, including total sleep time, sleep efficiency, and nocturnal awakenings, represent separate but related measures of sleep organization. In contrast, N3 sleep and slow-wave activity reflect deeper NREM sleep and synchronized cortical activity. Although these domains may overlap, improvement in one domain does not establish improvement in another. In particular, sedation alone should not be interpreted as evidence of enhanced slow-wave sleep.

Available human PSG studies suggest that mirtazapine may influence objective sleep architecture, including reported changes in N3 sleep or slow-wave sleep measures in selected populations [15-18]. However, these investigations do not establish a direct relationship between sedative potency and slow-wave sleep enhancement. Most available studies evaluated fixed doses rather than directly comparing insomnia-oriented lower doses with antidepressant-range doses, and the available literature remains insufficient to determine whether different dosing strategies produce distinct effects on slow-wave sleep. Observed PSG effects likely reflect the combined influence of mirtazapine's multiple pharmacologic actions, including histaminergic, serotonergic, and adrenergic effects [10-12]. Furthermore, PSG findings in patients with major depressive disorder should be interpreted within the context of broader treatment of depressive illness, as observed sleep changes may reflect both direct pharmacologic effects and possible improvement of depression-associated sleep disturbances [21,24]. In contrast, findings from healthy volunteer studies provide stronger evidence regarding direct pharmacologic effects of mirtazapine on sleep physiology, although their generalizability to clinical populations remains limited.

Depression-related sleep disturbances involve complex interactions among circadian regulation, monoaminergic signaling, and neural systems regulating wakefulness and sleep rather than a single abnormal sleep parameter [24,25]. Therefore, improvements in sleep observed during mirtazapine treatment should be interpreted differently depending on the study population: in clinical populations, sleep changes may reflect both treatment of depressive illness and direct pharmacologic effects, whereas healthy volunteer studies provide evidence regarding potential direct effects on sleep physiology independent of antidepressant response.

The relationship between mirtazapine dose, sedation, and slow-wave sleep highlights the complexity of pharmacologic modulation of sleep architecture. Although lower doses are clinically associated with greater sedation, sedative effects, sleep initiation, and objective enhancement of slow-wave sleep represent distinct physiologic outcomes. Current evidence supports a biologically plausible association between mirtazapine exposure and selected measures of sleep architecture, including reported changes in N3 sleep or slow-wave sleep in some studies; however, evidence regarding effects on quantitative slow-wave activity, dose dependence, and clinical significance remains limited and uncertain [10,12,15]. Future studies should directly compare different mirtazapine doses using standardized PSG methodology to determine whether insomnia-oriented lower doses differ from antidepressant-range doses in their effects on N3 sleep and slow-wave activity. Such investigations will clarify whether modulation of slow-wave sleep represents a clinically meaningful contributor to therapeutic response or simply a secondary physiologic consequence of mirtazapine’s broader receptor-mediated actions.

Limitations and future directions

Several limitations should be considered when interpreting the available evidence regarding mirtazapine and slow-wave sleep. First, the existing human polysomnographic literature is limited by relatively small sample sizes, heterogeneous study populations, variable treatment durations, comparator conditions, and differences in PSG methodology, which restrict the ability to draw definitive conclusions regarding the magnitude, reproducibility, and clinical significance of mirtazapine-associated changes in sleep architecture [13-18]. These differences are summarized in Table 3, which provides a study-level comparison of sample size, population characteristics, mirtazapine dose, treatment duration, comparator conditions, PSG parameters evaluated, and principal findings.

Additionally, many available PSG studies were conducted in patients with major depressive disorder, making it difficult to distinguish direct pharmacologic effects from normalization of depression-related sleep abnormalities. Although some investigations incorporated baseline PSG assessments and within-subject comparisons before and after mirtazapine exposure, the absence of placebo or healthy control conditions in several clinical studies limits the ability to determine whether observed changes represent direct medication effects or improvement of underlying depression-associated sleep disturbances. In contrast, placebo-controlled studies conducted in healthy volunteers provide stronger evidence regarding direct pharmacologic effects on sleep physiology, although their generalizability to clinical populations remains limited [13-18,21,24].

Although lower-dose mirtazapine is frequently used clinically because of its sedative properties, current evidence does not establish whether lower doses preferentially enhance slow-wave sleep or whether sedation and improvements in sleep continuity translate into meaningful increases in restorative sleep physiology. Importantly, sedative effects, reduced sleep latency, improved sleep continuity, and enhancement of slow-wave sleep represent distinct physiologic outcomes and should not be considered interchangeable.

Furthermore, available PSG findings reflect the integrated effects of multiple simultaneous receptor actions and cannot isolate the contribution of individual mechanisms, including histamine H1, alpha-2 adrenergic, or serotonergic receptor modulation [10-12]. Because mirtazapine acts across multiple receptor systems, observed changes in sleep architecture cannot determine whether specific receptor mechanisms are responsible for alterations in N3 sleep or slow-wave sleep-related measures. Whole-drug PSG observations therefore support potential relationships between mirtazapine exposure and sleep architecture but do not establish receptor-specific mechanisms underlying these changes [13-18].

Future investigations should focus on larger, well-controlled polysomnographic studies designed to clarify the relationship between mirtazapine dose, receptor-mediated effects, and slow-wave sleep regulation. Studies directly comparing lower insomnia-oriented doses with standard antidepressant doses should incorporate randomized, placebo-controlled designs, appropriate washout periods when crossover methodologies are used, standardized PSG acquisition and scoring criteria, quantitative EEG spectral analysis of slow-wave activity, and sufficient treatment duration to distinguish acute pharmacologic effects from longer-term changes in sleep architecture [13-18,20]. Such studies may help determine whether clinically observed differences in sedation correspond to measurable changes in N3 sleep or slow-wave activity.

Additional research incorporating diverse clinical populations and integration of PSG findings with neurobiological markers of sleep regulation may further clarify how mirtazapine's complex receptor profile influences sleep architecture and whether these effects contribute to therapeutic outcomes. Ultimately, determining whether modulation of slow-wave sleep represents a clinically meaningful mediator of antidepressant response or simply a secondary physiologic consequence of mirtazapine treatment will require carefully controlled studies capable of separating medication effects from underlying disease-related changes in sleep physiology.

## Conclusions

Mirtazapine possesses a distinctive pharmacologic profile that provides several biologically plausible pathways through which it may influence sleep regulation and sleep architecture, including histamine H1 receptor antagonism, alpha-2 adrenergic modulation, and serotonergic receptor effects. However, these mechanisms should be interpreted as potential contributors to sleep-related changes rather than established receptor-specific mechanisms responsible for alterations in slow-wave sleep. Human polysomnographic studies suggest that mirtazapine can produce measurable changes in objective sleep architecture; however, evidence specifically regarding N3 sleep and slow-wave sleep measures remains preliminary and mixed. Reported findings have varied across study populations, treatment durations, dosing strategies, comparator conditions, and methodological approaches, limiting conclusions regarding the consistency, magnitude, and clinical significance of these findings.

Although lower-dose mirtazapine is frequently used clinically because of its sedative properties, sedation should not be considered equivalent to enhancement of slow-wave sleep. N3 sleep, slow-wave sleep measures, and quantitative slow-wave activity represent related but distinct physiologic outcomes, and improvement in one domain does not necessarily indicate improvement in another. At present, modulation of slow-wave sleep should be viewed as a biologically plausible but unconfirmed secondary physiologic effect of mirtazapine rather than an established therapeutic mechanism.

Future studies incorporating randomized controlled designs, standardized polysomnographic protocols, quantitative EEG analysis, larger samples, longer treatment durations, and systematic evaluation of dose-dependent effects are needed to determine whether mirtazapine-associated changes in sleep architecture, including potential effects on slow-wave sleep, contribute meaningfully to clinical outcomes.
